# Stimulus uncertainty predicts serial dependence in orientation judgements

**DOI:** 10.1167/jov.22.1.6

**Published:** 2022-01-12

**Authors:** Geoffrey K. Gallagher, Christopher P. Benton

**Affiliations:** 1 School of Psychological Science, University of Bristol, Bristol, UK; 2 School of Psychological Science, University of Bristol, Bristol, UK

**Keywords:** serial dependence, orientation, Bayesian theories of perception

## Abstract

How is what you see influenced by what you saw? The visual system may use recent perception to inform responses to current stimuli. This can cause the perception of current stimuli to be attracted toward previous observations, an effect termed serial dependence. This misperception might well be useful in a noisy visual environment, where minor image distortions over time may not actually represent meaningful change. Previous work has suggested that Bayesian perceptual inference may underlie serial dependence. For this to be true, the relative uncertainty associated with both prior and current sensory input should be taken into account. In an experiment manipulating the level of noise present in orientation stimuli, we found an effect of current stimulus uncertainty on serial dependence. We found no good evidence for an effect of previous stimulus uncertainty. Our results provide only partial evidence for the Bayesian interpretation of serial dependence. Non-Bayesian models may provide a better account of the phenomenon.

## Introduction

Visual perception is contingent on overcoming noise in the visual signal. Environments are often dynamic, featuring noisy elements, such as rainfall or intermittent obstructions, which may hinder vision. Additionally, once a visual signal has reached the retina, neural noise in the visual system can corrupt the signal. Despite these sources of noise, we generally form stable visual representations of our surroundings. This might well be achieved by exploiting predictable stability in the environment (Dong & Atick, 1995; Girshick, Landy, & Simoncelli, 2011). One possible example of the visual system taking advantage of environmental regularities comes from an effect termed serial dependence (Burr & Cicchini, 2014; Fischer & Whitney, 2014).

Serial dependence is a visual misperception that can occur between very similar stimuli. When two stimuli are viewed in quick succession, and the differences between them are small enough, the second stimulus is often reported as being more similar to the first than it actually is (Burr & Cicchini, 2014; Kiyonaga, Scimeca, Bliss, & Whitney, 2017). Experiments by Fischer and Whitney (2014) demonstrated this effect in Gabor stimuli displaying different orientations. If the angles shown in successive stimuli were within a certain range of one another, perceptual conflation was shown to occur, where the orientation of the second stimulus was misreported as being closer to the orientation of the prior stimulus.

This phenomenon has since been found in many different areas of visual perception such as: numerosity (Fornaciai & Park, 2018), facial identity (Liberman, Fischer, & Whitney, 2014), facial age (Clifford, Watson, & White, 2018) emotional expression (Liberman, Manassi, M., & Whitney, 2018), attractiveness judgements (Van der Burg, Rhodes, & Alais, 2019; Xia, Leib, & Whitney, 2016), body size estimation (Alexi, Cleary, Dommisse, Palermo, Kloth, Burr, & Bell, 2018), aesthetics (Kim, Burr, & Alais, 2019), position (Bliss, Sun, & D'Esposito, 2017; Manassi, Liberman, Kosovicheva, Zhang, & Whitney, 2018), visual variance (Suárez-Pinilla, Seth, & Roseboom, 2018) and motion (Alais, Leung, & Burg, 2017).

The prevalence of serial dependence across visual categories suggests that it might be a general feature used to stabilize perception against noisy visual input. By “blending” the perception of two very similar stimuli viewed over short timescales, the visual system is relying on expected regularities in the environment to smooth over differences that are likely to be due to noise (Fischer & Whitney, 2014). Notably this effect does not occur when stimulus disparities are larger, and change is therefore more likely to reflect meaningful differences in the environment (Burr & Cicchini, 2014; Fischer & Whitney, 2014).

Although serial dependence may reflect a strategy to cope with noise in perceptual inference, for optimal perception it should also vary with stimulus reliability (Burr & Cicchini, 2014). Previous perception may be a useful guide for how to interpret current sensory evidence when there is some doubt surrounding what you are seeing, in this case greater weight should be given to prior perception rather than the current evidence (van Bergen & Jehee, 2019). However, if previous perception is less reliable, current evidence should be given more weight, and effects such as serial dependence should be downregulated. This approach approximates a Bayesian perceptual strategy, where estimates are weighted by their relative reliability (de Lange, Heilbron, & Kok, 2018; Kersten, Mamassian, & Yuille, 2004; Knill & Pouget, 2004).

Previous research has suggested uncertainty regarding current stimuli can affect serial dependence. Cicchini, Mikellidou, and Burr (2018) manipulated uncertainty by varying the characteristics of Gabor stimuli. Observers generally find judgements of orientation around cardinal axes easier (Girshick et al., 2011). Cicchini et al. (2018) found that Gabor stimuli oriented around the cardinal axes, where stimulus uncertainty is lower, produced reduced levels of serial dependence. Stimulus uncertainty was also manipulated by varying the frequency of Gabor gratings; higher frequency Gabors being taken to be more precise and, hence, less uncertain. Serial dependence was again found to scale with stimulus uncertainty; presenting observers with low frequency stimuli resulted in a greater tendency toward assimilative responses.

Expanding on this finding, van Bergen and Jehee (2019) found that the relative level of uncertainty dictated the strength of serial dependence. The authors developed a decoder which used the orientation preferences and correlated noise of voxels in functional magnetic resonance imaging (fMRI) data to build up a posterior distribution specifying the probability that any orientation produced the observed pattern of activation on a trial. The width of this posterior distribution quantified the degree of uncertainty, which was also found to correlate with behavioral variability in an orientation reproduction task. This was used to look at the impact of moving between different levels of stimulus uncertainty (i.e., high to low and low to high uncertainty). It was found that greater sensory uncertainty in the prior orientation stimulus relative to the current stimulus (as decoded from fMRI) reduced serial dependence, whereas reduced uncertainty in the prior orientation enhanced the effect. These results suggest that reliability of the current stimulus may be a factor that determines the strength of serial dependence in a manner consistent with Bayesian principles.

However, other work has produced results that do not fit with this Bayesian narrative. An experiment by Fritsche (2016) manipulated the signal-to-noise ratio of orientation stimuli to directly alter stimulus reliability. The authors examined the transition between stimuli with different noise levels. Their prediction was that serial dependence should be affected by the relative amounts of noise in current and prior stimuli. Their results differed from initial expectations; the transition from high to high noise stimuli resulted in greater serial dependence than the low to high transition. A similar result was also produced, contrary to author expectations, in the face stimuli experiments of Lidström (2019). When previous and current face stimuli were both degraded, serial dependence was enhanced. These results lie in conflict with Bayesian views of serial dependence, which would instead predict that relatively low noise in the prior stimulus should maximally enhance serial dependence.

Other work has also produced mixed results. The experiments of Ceylan, Herzog, and Pascucci (2021) manipulated uncertainty through differences in spatial frequency. Lower spatial frequency was used as a proxy for high uncertainty, in contrast to high spatial frequency stimuli, which are generally associated with lower uncertainty. This work suggested that only uncertainty in the current stimulus affected the strength of serial dependence. This was in opposition to the predictions of a Bayesian ideal observer model which predicts a reduced impact of low frequency prior stimuli. However, the authors note that low spatial frequency (i.e., higher uncertainty) stimuli tend to remain more stable across time in naturalistic environments. This may have acted against the predictions of the ideal observer model as this expected stability may also be taken into account when considering the reliability of previous stimuli (van Bergen & Jehee, 2019).

In the current experiment a similar procedure to that of Fritsche et al. was used to investigate the effect on serial dependence of noise in current and prior stimuli. The results of Fritsche et al. were contrary their own predictions. Given this unexpected pattern of results, we chose to collect new data using a sequence of stimulus movement which promoted a sense of continuity between stimuli. This was done to enhance serial dependence so that any effect of noise could be better distinguished. This work was undertaken operating under the hypothesis that, according to the predictions of a Bayesian model, serial dependence should be downregulated when a previous stimulus is noisy relative to the current stimulus, and enhanced when it is the current stimulus that is relatively noisy. Instead, we found that although uncertainty does affect serial dependence, this only appeared to apply to the current stimulus. We found no solid evidence that an attraction to prior orientations was affected by the level of uncertainty associated with the previous stimulus or response.

A truly Bayesian process would incorporate uncertainty in current and previous stimuli. The current results therefore leave the involvement of Bayesian processes unclear. The effect of ambiguity in the present stimulus may be more consistent with established ideas about serial effects based on decision processes (Treisman & Williams, 1984) or a previously described uncertainty-only model, also based on post-perceptual processes (Ceylan et al., 2021; Pascucci, Mancuso, Santandrea, Libera, Plomp, & Chelazzi, 2019).

## Materials and methods

This study was approved by the Psychological Science School Research Ethics Committee at the University of Bristol.

### Participants

Twenty participants took part in this experiment (15 female, ages 19–39, mean = 25.15, standard deviation = 4.91). All participants provided informed consent and were free to withdraw from testing at any time. Participants were paid £20 for taking part in this study. All participants had normal or corrected-to-normal vision.

### Stimuli

**Figure 1.**
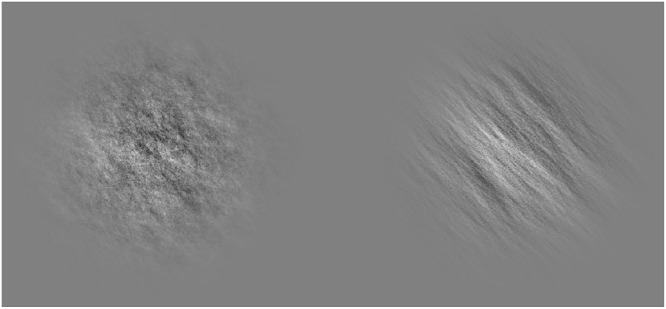
Examples of orientation stimuli used in experiment. *Left*, high noise, concentration of von Mises function set to 0.5. *Right*, low noise, concentration of von Mises function set to 4. Both stimuli show the same orientation.

Stimuli consisted of circular patches containing orientation information. On each trial stimuli were assigned a random orientation from 0° to 180°. A pattern of 1/f noise (Field, 1987) was generated by adjusting the Fourier amplitude spectrum of white noise in Fourier space. An orientation filter was then applied to this noise. The width of this filter was specified with a double angled von Mises distribution. The concentration of the von Mises distribution was set to two different levels to produce low noise stimuli (concentration = 4), which consisted primarily of orientation information near the specified random orientation, and high noise stimuli (concentration = 0.5), which included more information from other orientations (Figure 1). These noise levels were chosen based on prior testing on author G.G. to find a value at which the task was straightforward and a value at which the task became difficult but not overwhelmingly so. The binary choice task outlined below was used to validate these noise levels. The noise masks used between trials were produced in the same way as the experimental stimuli, but with the concentration of the von Mises function set to 0 to produce stimuli with no discernible orientation (the passband of the noise included all possible orientations). Although this carries the risk that participants could in some cases confuse noise masks and high noise orientation stimuli, because the noise masks contain no coherent orientation information, this confusion would only impact the perceived noise level of stimuli and not orientation determination. Stimuli and noise patches were contained in a 1.5° standard deviation Gaussian envelope.

**Figure 2.**
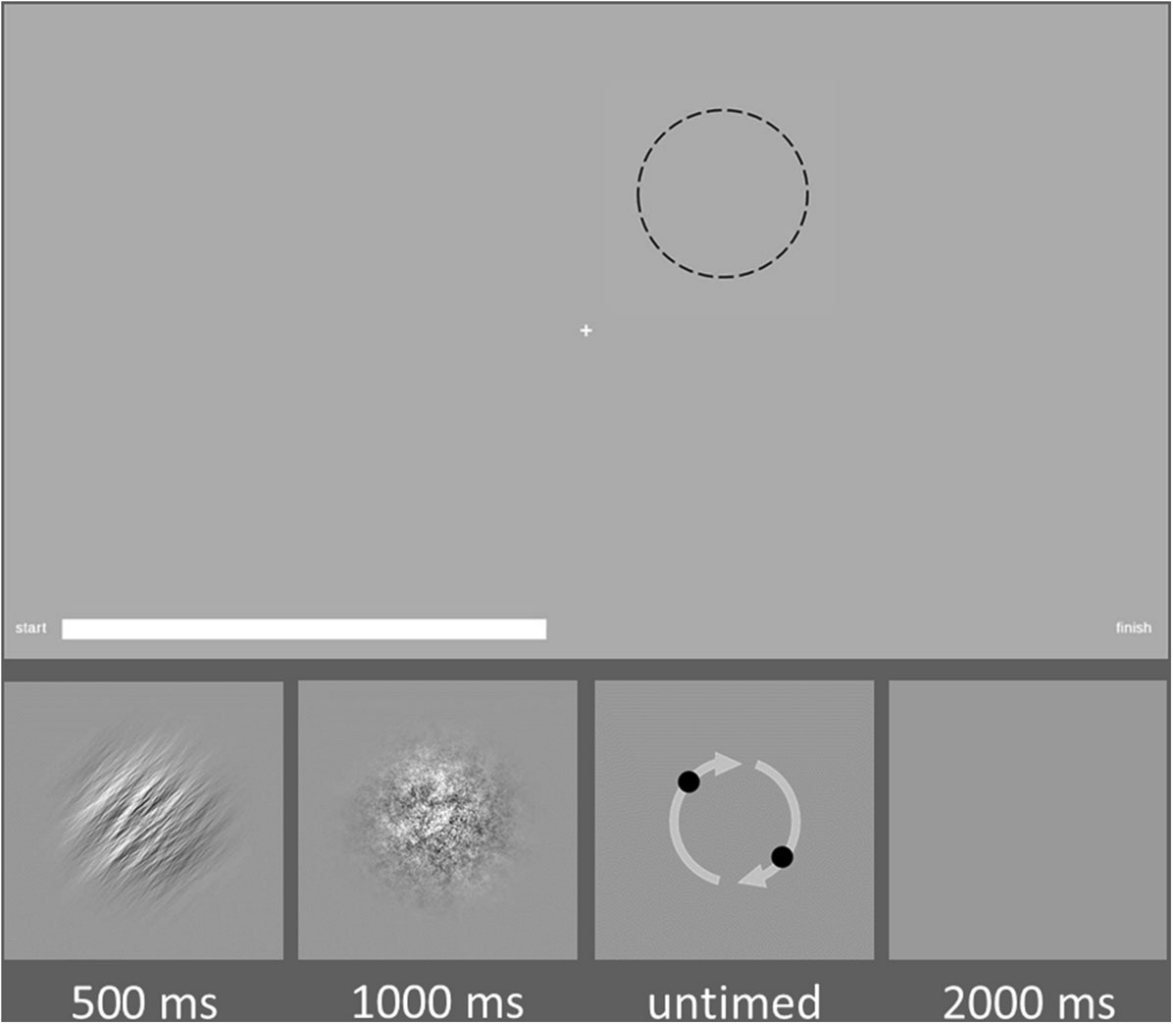
Typical trial sequence for the adjustment response portion of the experiment. Top image shows the screen as seen by a participant, stimuli (shown below) appear in the *dashed circle*. Participants saw a noisy orientation patch for 500 ms, followed by a noise mask for 1000 ms. An adjustment stimulus then appeared onscreen which allowed participants to make untimed responses to the initial stimulus. A grey screen followed the end of each trial and was displayed for 2000 ms. The next trial sequence appeared at a point 45 degrees counterclockwise of the previous trial sequence (*dashed circle*).

The response stimulus consisted of two black dots (width ∼1° visual angle) sitting on opposing poles of the area previously occupied by the orientation stimulus. The position of these dots could be rotated by the participant such that an imagined line connecting the dots would match the orientation of the experimental stimulus (See Figure 2 lower row, third panel). The initial orientation of the dots was random.

A white bar at the bottom of the screen indicated progress in each task, increasing in length as trials were completed. Participants were instructed to fixate on a central fixation cross (width 0.25° visual angle) for the duration of each experiment.

Stimuli were presented on a 24-inch VIEWPixx 3D lite monitor (VPixx Technologies) with a resolution of 1920 × 1080 and a refresh rate of 120 Hz. All experimental scripts were created in Matlab 2019b using Psychtoolbox. Psychophysics Toolbox for MATLAB (Brainard, 1997; Kleiner, Brainard, Pelli, Ingling, Murray, & Broussard, 2007; Pelli, 1997). Stimuli were viewed from approximately 57 cm in a darkened room.

### Procedure

Participants completed 821 trials of an adjustment task. Each trial presented participants with either a high or low noise stimulus of random orientation, which they were subsequently prompted to reproduce. The noise level of each trial was drawn from a random sequence designed to produce an even number of each noise level transition type (high noise to high noise, low to low, low to high and high to low) over the course of an experimental run.

Each trial followed the same format. First an experimental stimulus was presented for 500 ms. This was followed by a noise mask for 1000 ms to eliminate visual aftereffects. The response stimulus then appeared at the same location. After a participant response, a gray screen was then displayed for 2000 ms before the start of the next trial (trial sequence shown in Figure 2). This task took participants around an hour to complete.

Orientation stimuli, noise, and response stimuli appeared at points on a circle of radius nine degrees visual angle which enclosed a central fixation cross (see top panel of Figure 2). Each stimulus sequence appeared at a point 45° counterclockwise from the previous stimulus sequence. This circular path of stimuli around the screen was intended to promote serial dependence by providing a consistent object path, which has been shown to favor enhanced serial dependence (Liberman, Zhang, & Whitney, 2016). As noted above, a previous study (Fritsche, 2016) failed to find a relationship between stimuli/conditions that conformed to Bayesian expectations—we reasoned that increasing the signal strength would better allow us to describe any putative effects of uncertainty. Additionally, moving stimuli between spatial positions serves to minimize retinotopic adaptation (Afraz & Cavanagh, 2008).

**Figure 3.**
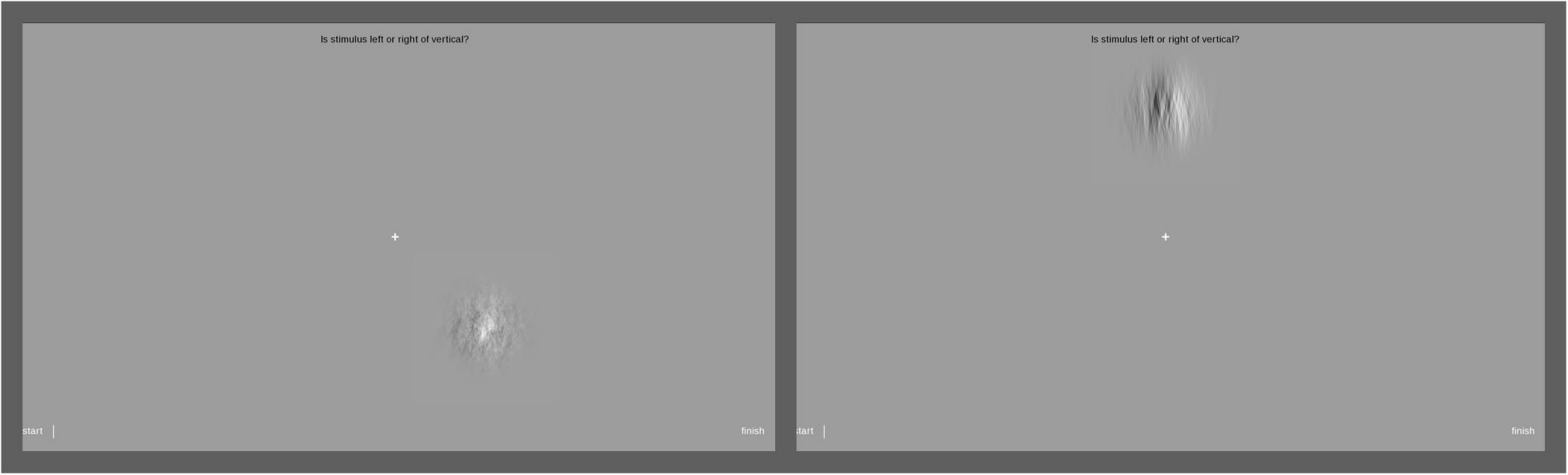
Consecutive trials in the binary choice task. *Left panel* shows a high noise stimulus; *right panel* shows low noise. Stimuli could appear randomly at any of the eight points (45° increments) around the central fixation cross. Stimuli remained onscreen until a response was made.

In another session in the weeks after the initial task, participants additionally completed a binary choice task. This task was designed to assess accuracy to confirm that the manipulation of noise levels did affect participants’ ability to determine stimulus orientation. Two blocks of this task were completed. In one block participants saw low noise stimuli and in the other high noise stimuli. Participants were asked to decide whether the stimulus displayed was oriented to the left or the right of an imaginary vertical line bisecting the stimulus. This task resembled the adjustment task in appearance. Participants saw stimuli at random points around the circular path taken by stimuli in the adjustment task (consecutive trials of this task shown in Figure 3). Displaying stimuli at random points was intended to minimize serial dependence by producing an inconsistent object path (Liberman et al., 2016). Stimuli stayed on screen until participants made a left or right response. Feedback text indicated correct or incorrect responses before the next stimulus appeared. Participants took around an hour to complete both blocks of the task.

Participants responded using a Microsoft Sidewinder controller. In the adjustment task the shoulder buttons were used to rotate the adjustment stimulus. Buttons on the right-hand side of the controller were used to switch between two rotation speeds and to confirm the angle of rotation to move onto the next trial. In the binary choice task, the shoulder buttons were used to indicate a left or right response, at which point the current trial ended. Participants were allowed to complete practice trials for each task until they were comfortable with the control scheme and instructions provided. Practice trial data were not included in analysis.

### Analysis

#### Binary choice task

For each participant, an integral of Gaussian curve was fit using probit analysis (Finney, 1971) to the plot of probability of a clockwise response as a function of stimulus orientation. This procedure was performed for both noise levels. The standard deviation (spread) of this function was taken to indicate sensitivity in participant response, with a high standard deviation indicating that participants found the task difficult.

#### Main task

The data gathered from the main adjustment task was subjected to two main forms of analysis. An analysis based on the technique of fitting a derivative of Gaussian curve to the data (Fischer & Whitney, 2014) and a “model-free” analysis based on estimating the average bias observed on trials (Samaha, Switzky, & Postle, 2019). These analyses were applied to investigate the relationship between prior and current orientation perception. The difference between successive trials was calculated in two different ways, being based on either prior stimuli or responses to prior stimuli (outlined below). Both stimulus- and response-contingent data were subjected to model based and model free analyses.

Data were subject to preprocessing to remove non-serial-dependence sources of response bias, in line with previous studies (Fritsche, Mostert, & de Lange, 2017; Pascucci, Mancuso, Santandrea, Libera, Plomp, & Chelazzi, 2019). To remove clockwise/counterclockwise response biases, for each participant the circular mean of their response errors was calculated, and this was then subtracted from the raw response error data (Fritsche et al., 2017). Errors greater than three standard deviations from the mean error of each participant were removed (Fritsche et al., 2017). This mean error was calculated separately for low- and high-noise trials. This procedure resulted in an average of 0.76% of trials being removed for each participant.

The extent of serial dependence is thought to be determined by the difference between the stimulus observed on the current trial, and the stimulus perceived on the previous trial (Fischer & Whitney, 2014). This can be calculated as the difference between the current stimulus and either the previous stimulus, or the previous response. There should be an association between these values and observer perception of stimuli, but we would never expect this to be a perfect correspondence. However, the relationship between perception and stimulus should be greatly affected by stimulus noise, whereas the relationship between perception and response should not.

We would expect increases in stimulus noise to increase the variability of perception. When viewing a noisy 90° orientation stimulus, an observer might respond that it does look like it is oriented at ninety degrees. On viewing another 90° stimulus, the observer might decide that this time it looks more like a 70° stimulus, and this is again revealed in their response. This variation indicates that the stimulus value itself might not accurately represent the perceived value, and assuming that it does introduces noise into the relationship between current and previous trials.

If we are conditioning on the stimulus as a proxy for the percept, then this measure (the difference between current and previous stimulus) becomes noisier as stimulus noise increases. If serial dependence is some function *f(x)* of the difference between current stimulus and previous percept, and if we can think of the stimulus noise as a Gaussian function *g(x)*, then the resultant observed relationship will be described by the convolution of the two (g(x) * f(x)). What this tells us is that, as stimulus noise increases, we would expect the observed function to decrease in amplitude and increase in spread. Assuming that observers are making an honest attempt to report their perception, then taking the response as a measure of perception avoids this problem (Fritsche, 2016). So, in experiments where the focus is on the effect of stimulus variability, conditioning upon previous stimulus is a poor choice; conditioning upon previous response is to be preferred. Studies that have looked at stimulus uncertainty have used both stimulus-contingent (Ceylan et al., 2021) and response-contingent (Fritsche, 2016) approaches. In the present study we performed both stimulus- and response-contingent analyses.

A technique previously used by Pascucci et al. (2019) was implemented in order to avoid the oblique response bias found in this type of response-based analysis (Fritsche, 2016). A sum of sin waves model was fit to the plot of participant errors against orientation (fitting performed using MATLAB “fit” function with “Normalize” = “on” as per the methods of Pascucci et al.). This model was used to capture orientation-dependent biases in participant responses. Errors predicted from this model were then removed, leaving data free of orientation-dependent response error. Individual models were fit for each participant. Because uncertainty is known to produce differences in oblique perception (Tomassini, Morgan, & Solomon, 2010), high- and low-noise trials were modeled separately. Pascucci et al. downweighted errors greater than three standard deviations from the mean, whereas in the current study similar values were removed in the previous stage of processing. This model was applied exclusively to response-contingent analyses. With stimulus-contingent analysis there is a risk that this correction could induce artifacts caused by known uncertainty-dependent differences in orientation biases (Tomassini et al., 2010).

##### Model-based analysis

A derivative of Gaussian (DoG) curve was fit to the moving average (window size of 20°) of participant errors conditioned on the difference between current trial and the previous trial (for both stimulus- and response-contingent differences). The DoG curve was identified by Fischer and Whitney (2014) as describing the form of serial dependence. The DoG is defined as follows:

(1) $$y=xawce^{-{\left(wx\right)}^{2}}+b$$

where:

*y -* participant response error
*x -* relative orientation of the previous trial
*a* - amplitude of the curve
*w* - curve width
*c -* constant: √2/*e*^−0.5^.
*b* - baseline

Parameter *a* represents the strength and direction of serial dependence, showing the peak response error observed (values reported represent the half amplitude height of the curve from 0). Parameter *w* describes the stimulus range over which serial dependence is occurring. The value of *w* was constrained between 0.02 and 0.2.

In line with the analysis methods of Fischer and Whitney (2014) permutation tests were performed to assess the amplitude of the DoG curve. For a random subset of participants, the sign of the data was flipped, and curve fitting was performed on this artificial dataset. This was repeated 10,000 times; *p* values were calculated by taking the percentage of permutations that produced values of *a* of greater magnitude than the observed value of *a*.

To test the difference between conditions permutation tests were performed on the difference in DoG amplitude in a procedure similar to one employed by Fritsche (2016). For each pairwise comparison, condition labels were randomly swapped within participants and DoG curves were fit to the pooled moving average (moving average window size of 20) of each condition. The amplitude of the DoG curve for one permuted condition was then subtracted from the other. This procedure was repeated 10,000 times. As *p* values we report the proportion of values in the resulting distribution greater in magnitude than the observed difference.

##### Model-free analysis

Model-based analyses may produce spurious fits to data. An assumption in fitting the DoG curve is that, outside of the range of serial dependence, participant errors fit onto a flat line. This assumption can often be incorrect. The DoG model has previously been observed to produce poor fits to data in the presence of “peripheral bumps,” where attractive biases instead become repulsive at large orientation differences (Bliss et al., 2017).

As noted above, a model-free analysis was also applied to our data (Samaha et al., 2019). The median error for each participant was calculated separately for trials where the difference between stimuli was either between 0° and 45° or 0° and −45° degrees. This range, previously identified by Samaha et al., is where we expect to observe serial dependence, while excluding sidebands. The median error value for the negative differences was subtracted from the median for positive differences to produce one value that indicated attractive (positive values) or repulsive (negative values) serial biases for each participant. Bias values were compared using a two-way repeated measures analysis of variance (ANOVA) to determine the effect of previous and current noise levels, as well as their interaction. This model-free analysis was applied to both stimulus- and response-contingent data. All statistical analyses were carried out in Matlab with the exception of ANOVA, which was performed using SPSS.

## Results

Five participants were excluded from the serial dependence analysis due to poor performance. These participants showed a correlation between stimulus and response of less than 0.5, indicating that they were either responding without care, or were finding the task unusually challenging. Participant performance was treated separately for each task; participants excluded from serial dependence task analysis were not necessarily excluded from the binary choice task analysis, and vice versa. Three participants were excluded from analysis of the binary choice task because they produced response values far outside of the range of other participants. For these participants the standard deviation (spread) of the fitted cumulative normal was above 60°, whereas those of the remaining participants ranged between 1° and 9°. One other participant was excluded from binary choice analysis for misunderstanding the task.

### Binary choice task

Performance on the binary choice task confirmed that participants found it more difficult to discern the orientation of the high noise stimulus (standard deviation of cumulative normal = 25.24 for high noise, 4.23 for low noise, significant difference according to permutation test of differences between the two conditions *p* < 0.001, Cohen's *d* = 1.02). Consistent with these results, participant accuracy was lower for high noise stimuli in the adjustment task (Mean error for high noise stimuli 23.90°, mean error for low noise stimuli 16.22°, *t*(19) = 4.57, *p* < 0.001, paired *t*-test, Cohen's *d =* 1.02).

Previous work has used the standard deviation of cumulative Gaussian functions to infer the reliability of stimuli on each trial and determine the extent of serial dependence between any two stimuli according to an ideal observer model (Cicchini et al., 2018). The binary choice task in this experiment was designed to avoid serial dependence effects; stimuli appeared at random points and remained onscreen until a choice was made. In contrast, the adjustment task was open to factors such as post-perceptual effects due to delay periods. Given these differences between the tasks, variability inferred from the binary choice task might not be useful in predicting response variability in the main adjustment task, limiting how useful it can be in extrapolating uncertainty effects on serial dependence. Nevertheless, the binary choice task does suggest that our noise manipulation induced greater uncertainty in participants.

### Model-based analysis

#### Analysis of the four transitions

**Figure 4.**
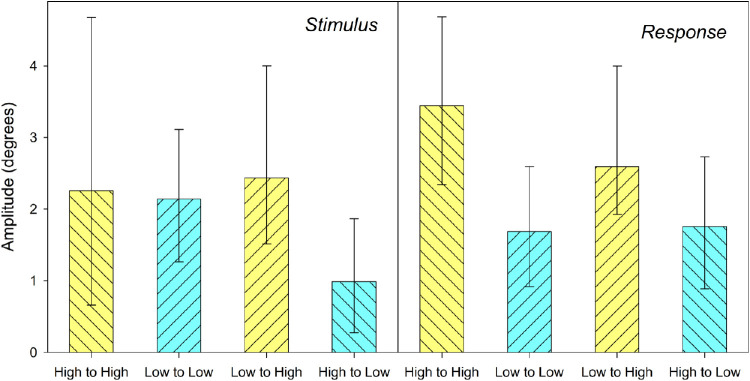
DoG amplitudes for each transition. *Left graphs* show amplitude where difference is based on difference between current stimulus and previous response. *Right graphs* show data based on previous stimulus. *Error bars* represent 95% confidence intervals.

**Figure 5.**
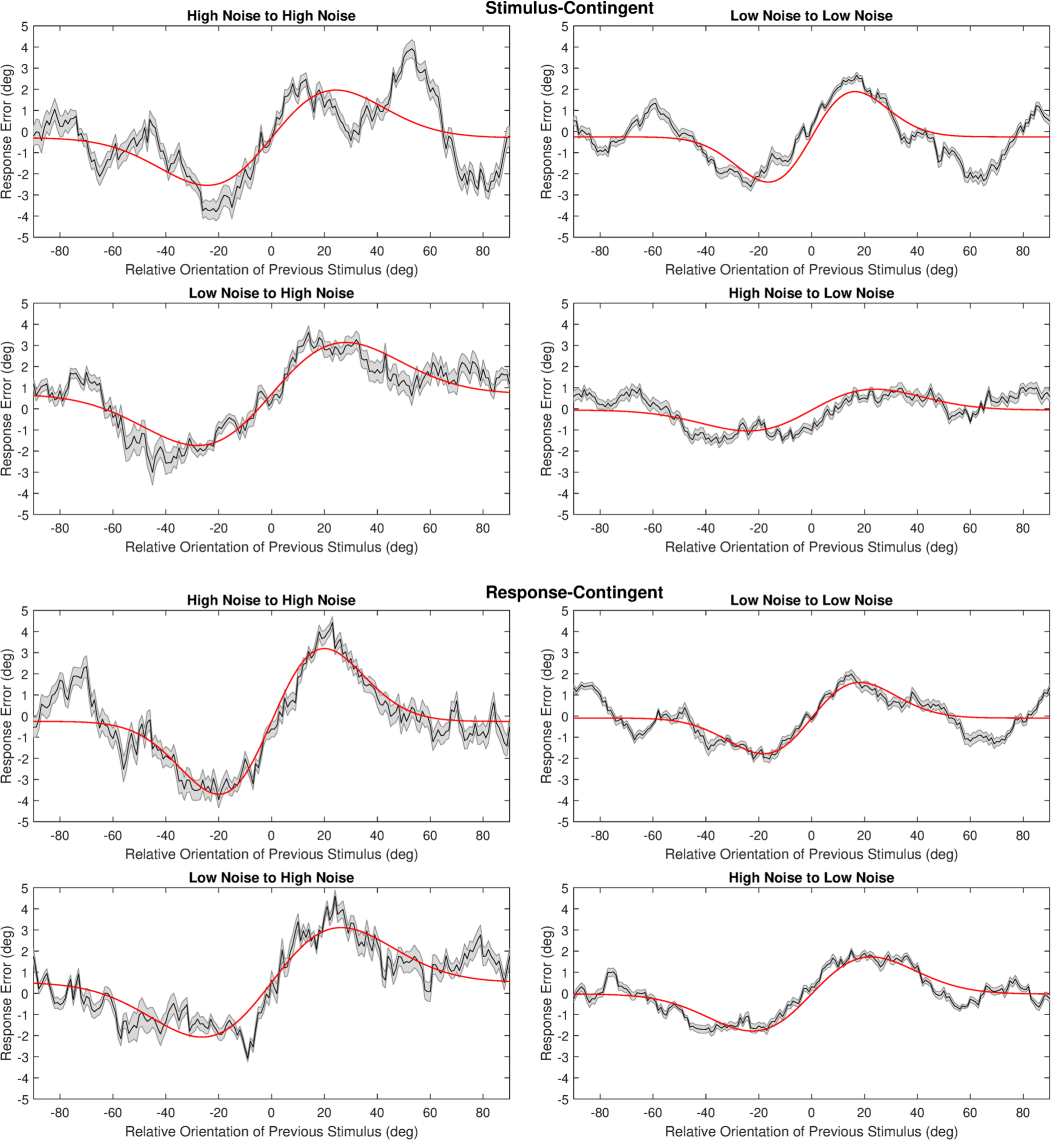
Transition conditions for pooled data. *Red line*, DoG fit; *black line*, moving average. *Gray shading* indicates standard error. Top four graphs show stimulus-contingent data. Bottom four graphs show the same transitions for response-contingent data.

Stimulus-contingent analysis produced positive amplitude values (see Figures 4 and 5), consistent with assimilative effects; however, the values for high noise and high noise to low were nonsignificant (Low noise to low noise, *a* = 2.14°, *p* = 0.001, Root Mean Squared Error = 0.82; High noise to high noise, *a* = 2.25 degrees, *p* = 0.098, RMSE = 1.27; High noise to Low, *a* = 0.98 degrees, *p* = 0.059, RMSE = 0.54; Low noise to High, *a* = 2.4 degrees, *p* = 0.006, RMSE = 0.7). As a form of control analysis, the DoG fitting procedure for stimulus-based analysis was applied to participant errors conditioned on the relationship between orientation in current and future trials (t+1 analysis). No meaningful relationship should be observed, barring precognition. As expected, this analysis produced no significant results (all DoG amplitudes, in all conditions, *p* > 0.10).

Similarly, response-contingent analysis also yielded positive DoG amplitude values, however in this case all were significant (Low noise to low noise, *a* = 1.68 degrees, *p* = 0.003, RMSE = 0.56; High noise to high noise, *a* = 3.44 degrees, *p* < 0.001, RMSE = 0.77; High noise to Low, *a* = 1.7 degrees, *p* = 0.003, RMSE = 0.43; Low noise to High, *a* = 2.59 degrees, *p* = 0.005, RMSE = 0.78).

#### Removing the oblique response bias

**Figure 6.**
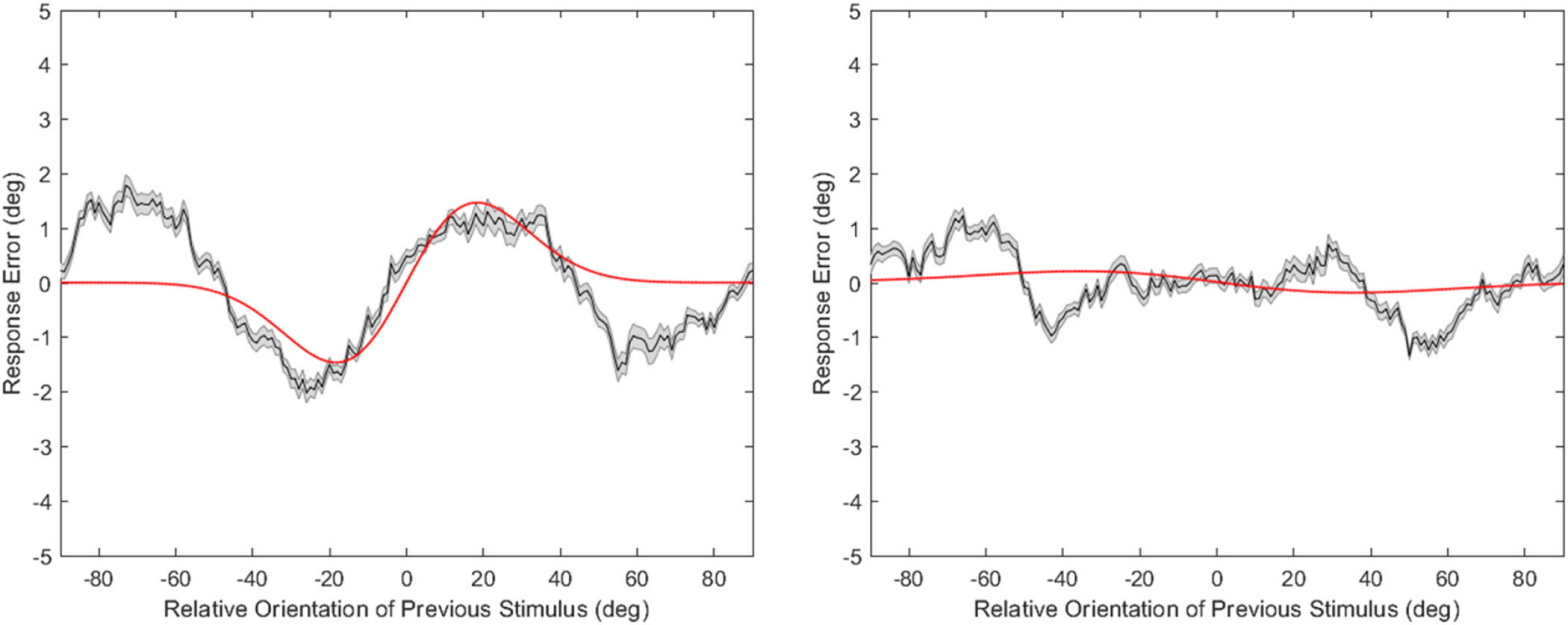
Alternate flip trial data pooled across transitions before and after correction. *Left-hand graph* shows data subject to the flip trial analysis before correction for the oblique response bias. *Right-hand graph* shows the same analysis with the oblique bias correction technique applied.

For response-contingent analysis, an oblique response bias may produce spurious serial dependence (Fritsche, 2016; Pascucci et al., 2019). To remove this effect, we applied a corrective procedure to our data as described above (results of this corrective procedure can be seen in Supplementary Figure S1). To confirm that this was successful, we ran another analysis with the aim of removing the temporal relationship between subsequent responses (alternate-flip-trial analysis). For each participant the order of even-numbered trials was inverted. If a participant completed 100 trials, trial 2 was swapped with trial 98, 4 with 96, 6 with 94, and so on. This removes any correlation between responses and response errors on paired trials. Because the oblique response bias is expected to apply on individual trials, we should still see serial dependence in uncorrected inverted response data but not in corrected data. This is exactly what we observe (see illustrative graph Figure 6), the alternate-flip-trial analysis shows several significant results before correction (high to high and low to low noise transitions *p* < 0.05, all data pooled across transitions *p* < 0.01,) and no such effect post-correction (all *p* values in all transitions, > 0.1).

#### Comparisons between transitions

The majority of differences between transitions were revealed to be non-significant for analysis based on both previous stimulus and previous response (*p* > 0.05, see Table 1). For stimulus-contingent DoG analysis the difference between high to low and low to high transitions was significant (*p* < 0.05). Response-contingent analysis yielded significant differences for the transitions between high to high and low to low noise, as well as high to high and high to low. Although these results are suggestive of differences between conditions, the width values associated with many iterations of model fitting were equal to the predetermined limits (0.02, 0.2). This indicates that the model fitting algorithm may have struggled to adequately fit the DoG function during the resampling procedure. This may be due to variability in portions of the response function that are not adequately described by the DoG driving variability in curve fits during the permutation procedure. Our data include clear sidebands that lie outside the central region accounted for by the DoG function (see Figure 5).

**Table 1.** *P* values for permutation testing of difference between transition conditions. Top values in each cell are derived from analysis based on previous stimulus. Lower values are derived from analysis based on previous response.

	High to High	Low to Low	Low to High	High to Low
High to High		.92	.87	.30
		.04	.18	.03
Low to Low	.92		.66	.06
	.04		.13	.88
Low to High	.87	.66		.04
	.18	.13		.13
High to Low	.30	.06	.04	
	.03	.88	.13	

#### Model-free analysis

**Figure 7.**
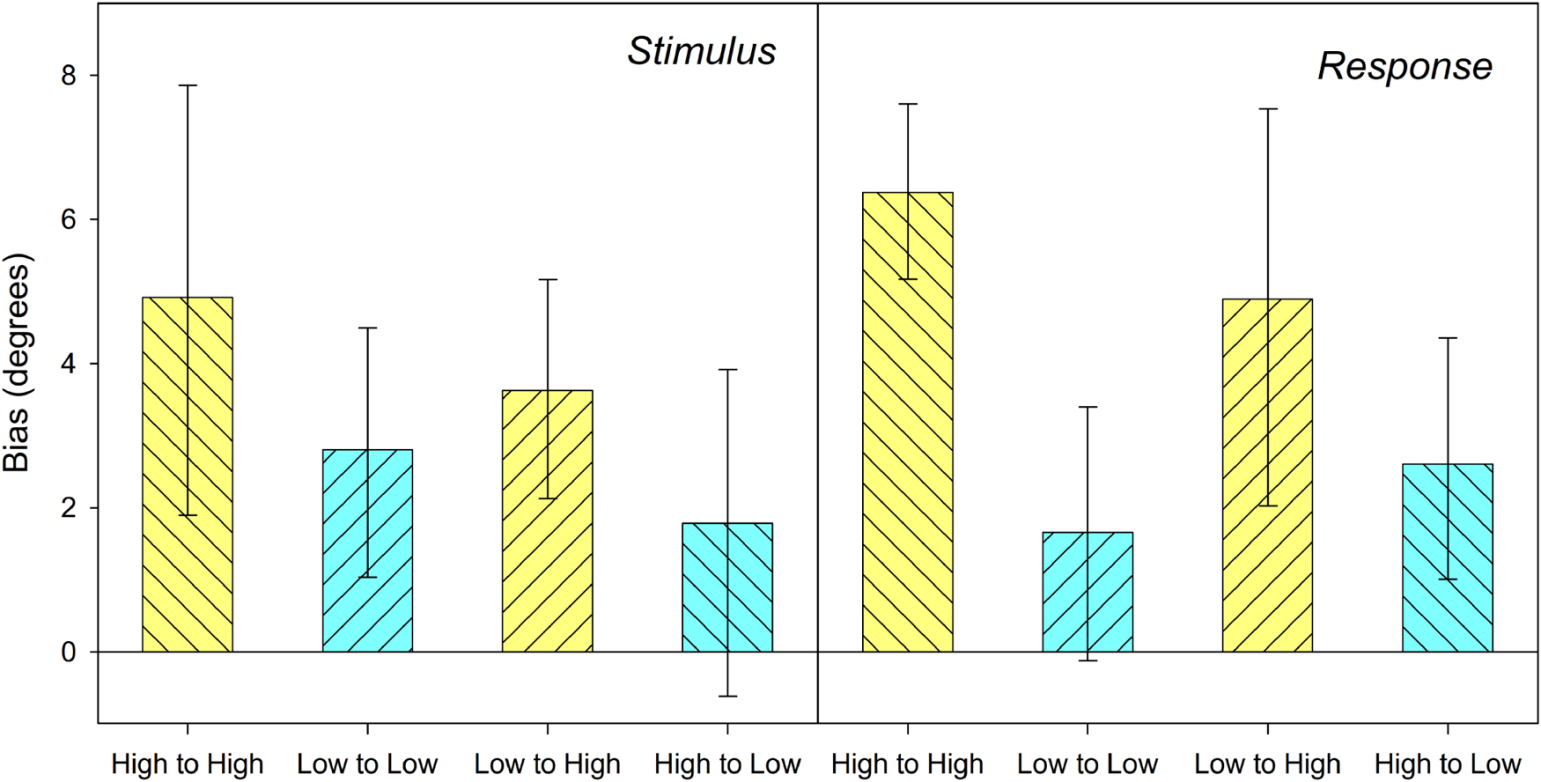
Model free biases. *Left graphs* show biases where difference is based on difference between current stimulus and previous response. *Right graphs* show data based on previous stimulus. *Error bars* represent 95% confidence intervals.

As with the model-based analyses, the model-free approach was carried out for orientation differences between trials based on previous stimuli and previous responses (see Figure 7). For stimulus-based analysis, calculation of the orientation difference from the previous trial used previous stimulus values whereas for response-based analysis the previous response was used (with the same sum of sin model residualization as was applied for DoG analysis). For both methods the overall relationship between transition conditions was the same. A two-way repeated measure ANOVA, with factors of previous stimulus and current stimulus, each with two levels (low noise and high noise), was carried out to determine the effect of noise in the current and previous stimuli on participant serial biases. This analysis suggested that the effect of noise was limited to the current stimulus (*F*(1,14) = 4.68, *p* = 0.048 for stimulus-based analysis, *F*(1,14) = 15.04, *p* = 0.002 for response-based analyses) and that noise in the previous stimulus did not contribute to the differences between conditions (*F*(1,14) = 0.03, *p* = 0.864 for stimulus-based analysis, *F*(1,14) = 1.65, *p* = 0.220 for response-based analysis). The interactions between current and previous noise were also not significant (*F*(1,14) = 0.64, *p* = 0.439 for previous stimulus, *F*(1,14) = 0.124, *p* = 0.730 for previous response).

## Discussion

We used orientation stimuli incorporating two different levels of noise to investigate the role of stimulus uncertainty in serial dependence. Participants were asked to reproduce the orientation of observed stimuli presented in succession. Participant orientation reports were consistently attracted toward the stimuli observed on the immediately previous trial. We observed that uncertainty in the presently observed stimulus can affect the scale of serial dependence. Using a model-free analysis, we found that higher uncertainty in the stimulus currently being observed can enhance the scale of this attractive effect; participant responses appeared to be more heavily biased toward previously observed stimuli when the current stimulus incorporated a higher level of noise. These findings support the idea that serial dependence is responsive to uncertainty in the current stimulus; however, we did not find similar evidence for an effect of uncertainty in prior stimulus.

Previous experiments have suggested that serial dependence may be enhanced under conditions associated with uncertainty (Ceylan et al., 2021; Cicchini et al., 2017; Cicchini et al., 2018; Manassi et al., 2018). These studies manipulated uncertainty through variation in stimulus properties known to affect the noisiness of the percept being judged (contrast, spatial frequency, cardinality), with these variations serving as a proxy for uncertainty. In the current experiment we used a direct manipulation of the uncertainty inherent in the judged percept; altering the orientation bandwidth of stimuli allowed variation in the range of orientation information presented (Beaudot & Mullen, 2006). An experiment by Fritsche (2016) used similar experimental stimuli. Their study produced results inconsistent with other experiments that document effects of uncertainty (Cicchini et al., 2018; Lidström, 2019). These latter experiments support a Bayesian narrative that suggests that serial dependence may optimally weight the reliability of stimuli (Cicchini et al., 2018).

The basic idea behind this is that for serial dependence to be useful in everyday perception, consideration of the strength of evidence for prior and current stimuli makes sense. If the current stimulus is ambiguous, it may be logical to take into account recent, more reliable perception when interpreting the current sensory evidence. Conversely, reliance on the recent past is not optimal when the evidence for current stimuli is stronger than the evidence for prior perception. In this case the better solution might involve attaching greater weight to current sensory representations rather than incorporating information from recent perception.

A number of studies have provided evidence that serial dependence may be responsive to uncertainty in a way that conforms to these Bayesian predictions. Work by van Bergen and Jehee (2019) reported a pattern of results proposed to be fully in line with Bayesian theories of perception. The authors found that greater sensory uncertainty in the prior orientation stimulus relative to the current stimulus (as decoded from fMRI) reduced serial dependence, whereas reduced relative uncertainty in the prior enhanced the effect. Similarly, the results of Cicchini et al. (2018) suggested a Bayesian pattern of serial dependence where transitions between uncertain stimuli showed greater serial dependence than transitions between relatively reliable stimuli.

The current results stand in contrast to these earlier findings. Although we observed an effect of uncertainty in the present stimulus that is consistent with these studies, the lack of evidence for an effect of uncertainty in the previous stimulus means that the Bayesian narrative is not fully supported by our results. On the other hand, the current results are consistent with the recent findings of Ceylan et al. (2021), who manipulated stimulus uncertainty through differences in spatial frequency. Lower spatial frequencies are associated with greater orientation uncertainty. The authors exploited this by using high- and low-frequency stimuli as proxies for low and high uncertainty, respectively. The authors found that the strength of serial dependence was dependent only on uncertainty in the current stimulus, in contrast to the predictions of a Bayesian model.

Ceylan et al. (2021) propose that this inconsistency with the findings of Cicchini et al. (2018) and van Bergen and Jehee (2019) is due to the fact that all possible stimulus noise transitions were not compared in those two studies. Ceylan et al. (2021) note that the study by Cicchini et al. only looked at the transitions between low- or high-noise stimuli (i.e., low to low noise or high to high noise in the current study). In contrast, the study by van Bergen and Jehee did look at the transitions between low and high noise stimuli but did not compare these to transitions between stimuli that produced the same level of uncertainty. This leaves open the possibility that in both of these studies uncertainty in the current stimulus alone was dictating the strength of serial dependence.

A persistent issue that is often raised in studies of serial dependence concerns the underlying basis of this effect. Serial dependence could be the product of early sensory processes (Fischer & Whitney, 2014) or it could in fact arise from postperceptual top-down mechanisms (Fritsche et al., 2017). Although the approach taken in the experiments of Cicchini et al. (2018) was agnostic with regards to the underlying basis of serial dependence, improvements in perceptual efficiency were taken as an indication that this process may apply at the level of underlying sensory representations. The work of Fischer and Whitney also framed serial dependence as an effect capable of producing genuine perceptual changes (Fischer & Whitney, 2014).

Alternatively, serial dependence may be implemented through the interaction of early visual areas with higher areas in the cortex (Vilares et al., 2012). Top-down decision effects have been suggested as a non-perceptual cause of serial dependence (Fritsche et al., 2017). Specifically, Fritsche et al. proposed that serial dependence might occur because of assimilation between current and previous decisions taking place at a postperceptual stage as opposed to a genuine merging of perceptions. Similarly, Akaishi, Umeda, Nagase, & Sakai (2014) coined the phrase “decision inertia” to describe the way that previous decisions may be repeated, a behavior that may be based on a preference for consistency (Alós-Ferrer, Hügelschäfer, & Li, 2016). Decision inertia could produce similar outcomes to those predicted by perceptual serial dependence. Can decision effects account for the pattern of responses found in our experiment?

Work investigating decision effects makes predictions about the impact of noise in the current stimulus. Ambiguity in the current stimulus has previously been suggested to cause a change in decision strategy (Petzold & Haubensak, 2001; Treisman & Williams, 1984). Treisman and Williams suggested that participants tend to repeat their previous response when unsure of the current stimulus. If serial dependence represents an attempt to repeat the previous decision, then we might therefore expect to see greater serial dependence when the current stimulus is ambiguous. This is exactly what we see in the current data; serial dependence is enhanced when the current stimulus is associated with greater uncertainty. This suggests that the serial effect observed could be reducible to something as simple as participants attempting to reproduce the previous stimulus when unsure on the current trial.

More complex decision-based effects have also been proposed. Pascucci et al. have suggested that a decision-weighted decoding of sensory input may instead occur (Ceylan et al., 2021; Pascucci et al., 2019). This more nuanced model involves a selective top-down weighting of the output of low-level sensory channels based on which channels have been recently informative. Responses indicating the presence of recently observed stimuli are given more weight because their continued activity can safely be assumed to indicate the continued presence of similar stimuli. This weighting is applied across channels and so can produce an attraction toward previous responses rather than strict response repetition. Crucially, in this model, the influence of the previous trial is inversely weighted by the variability associated with the current stimulus; variability in the previous stimulus having been discarded (Ceylan et al., 2021). The model therefore predicts an effect of variability in the current stimulus, but not in the previous stimulus. Ceylan et al. (2021) suggested that this postperceptual model provides a better account of the impact of stimulus uncertainty on serial dependence than a Bayesian model. In addition, this model, while based on postperceptual factors, does leave open the possibility of genuine perceptual change (Pascucci et al., 2019), something not necessarily predicted by response repetition.

The current experiment does not rule out either perceptual or decision-based accounts of serial dependence. However, results from Ceylan et al. (2021) suggest that, at least for orientation stimuli, serial dependence may arise from higher level sources. The authors found serial dependence between Gabor patches and orientation defined by the axis of symmetry of dot patterns (which requires higher level processing of symmetry). Serial dependence occurring beyond low-level representations suggests the possibility of postperceptual decision effects being involved in this process.

Uncertainty-based decision effects (Treisman & Williams, 1984) are consistent with the effect of current stimulus noise observed in the current data. However, nothing in the current experiments rules out concurrent contributions of perceptual and decision effects. Although explanations invoking low-level perceptual changes (Cicchini et al., 2017; Fischer & Whitney, 2014) or higher-level decision effects and working memory influences (Bliss et al., 2017; Fritsche et al., 2017; Pascucci et al., 2019) may appear to be in opposition, findings suggesting serial effects at different levels of the perceptual inference process could just demonstrate the utility of previous information in determining perception. More generally, if relying on recent information is useful in determining the current state of the world, then we might expect this strategy to be used in perceptual, decision, and memory processes.

An issue with any manipulation of uncertainty is that it tends to come with a concurrent change in response/percept variability. As we describe previously in our analysis section, this variation indicates that the stimulus value itself might no longer accurately represent the perceived value, and this can cause the appearance of reduced serial dependence. This problem of response/percept variability in previous stimuli can be overcome using a response-contingent analysis (Fritsche, 2016). Taking the previous response as an account of observer perception eliminates this source of extraneous variation, which would otherwise act as a confound. Response/Percept variability can specifically cause the appearance of reduced serial dependence. Our main finding, of an observed increase in serial dependence for current high noise stimuli, is therefore unlikely to be driven by response/percept variability.

Notably this effect of response variation could apply to any manipulation of uncertainty, including changes in spatial frequency that have previously been used (Ceylan et al., 2021; Cicchini et al., 2018). This may mean that any manipulation of stimuli that promotes variability carries this confound. Response-contingent analysis should ideally be applied in any experiments testing the effects of uncertainty on serial dependence.

For stimulus-contingent analysis, the misleading effects of response variability could potentially lead to the conclusion that previous stimulus uncertainty was capable of modulating serial dependence. Despite this, the current work, as well as previous stimulus-contingent studies (Ceylan et al., 2021), has failed to provide evidence of an effect of noise in the previous stimuli. However, an influence of previous uncertainty still cannot be ruled out, because it remains possible that other effects associated with noise could actually mask any impact of previous stimulus noise. Bosch, Fritsche, Ehinger, and Lange (2020) suggest that previous stimuli exert a concurrent repulsive effect that is strongest with greater evidence strength. This could potentially act in opposition to any noise-dependent augmentation of serial dependence, canceling out any clear effect of previous stimulus uncertainty.

Alternatively, to account for the lack of an effect of previous stimulus uncertainty, we can appeal to Bayesian processes that incorporate stimulus volatility. To accurately predict the environment, previous Bayesian models of serial dependence have incorporated additional priors that model the probability of stimulus change to provide an account of the role of uncertainty in serial dependence (Fritsche et al., 2020; van Bergen & Jehee, 2019).

In the current experiment stimulus orientation was generated randomly meaning there was no actual relationship between stimuli and any orientation similarity was coincidental. If observers can roughly quantify the probability of change in sequential stimuli, then this could also be factored into how much weight they give to prior observations. If this form of temporal uncertainty, or volatility, is high enough, it might wash out any effect of previous stimulus uncertainty. Essentially, the effect of temporal uncertainty might be high enough that any contribution of previous stimulus uncertainty is negligible in comparison. This might be tested experimentally using stimuli with a perceived low or high probability of change in tandem with an associated stimulus reliability. For now, all we can say is that uncertainty in the current stimulus is capable of affecting serial dependence, regardless of whether there is an effect of previous uncertainty.

Our model-free analysis, which makes fewer assumptions about the data, revealed an effect of stimulus uncertainty that was not apparent in the DoG analysis. With regard to the latter, Figure 5 shows noticeable variability in response errors that is not well modeled by the DoG curve. In particular, our data exhibited response errors at large orientation differences where the DoG function predicts no such errors, an issue previously identified by Bliss et al. (2017). This means that during permutation testing, these response error sidebands likely caused poor model fits to the data, hiding the significant effect revealed by model-free analysis. Although previous work has used an alternative model for data that exhibits these issues, this alternative model was also not always a good fit to data (Bliss et al., 2017). The model-free analysis may well be the best solution for data that do not conform well to the expectations of more complex models.

In conclusion, our results demonstrate that stimulus uncertainty can affect the magnitude of serial dependence; this effect appears to be limited to the effect of uncertainty in the current stimulus. Although it remains possible that uncertainty in the previous stimulus can affect serial dependence, evidence for this is lacking. The current research is in line with previous results that also demonstrate an effect of current stimulus uncertainty (Ceylan et al., 2021). Evidence of effects of uncertainty in prior stimuli in concert with uncertainty in current stimuli is necessary to confirm a Bayesian perspective of serial dependence.

## Supplementary Material

Supplement 1
