# Supplementary figures and images for: Stimulus uncertainty predicts serial dependence in orientation judgements
Source: J Vis. 2022 Jan 12;22(1):6. doi: 10.1167/jov.22.1.6 (PMC8762691; doi:10.1167/jov.22.1.6)

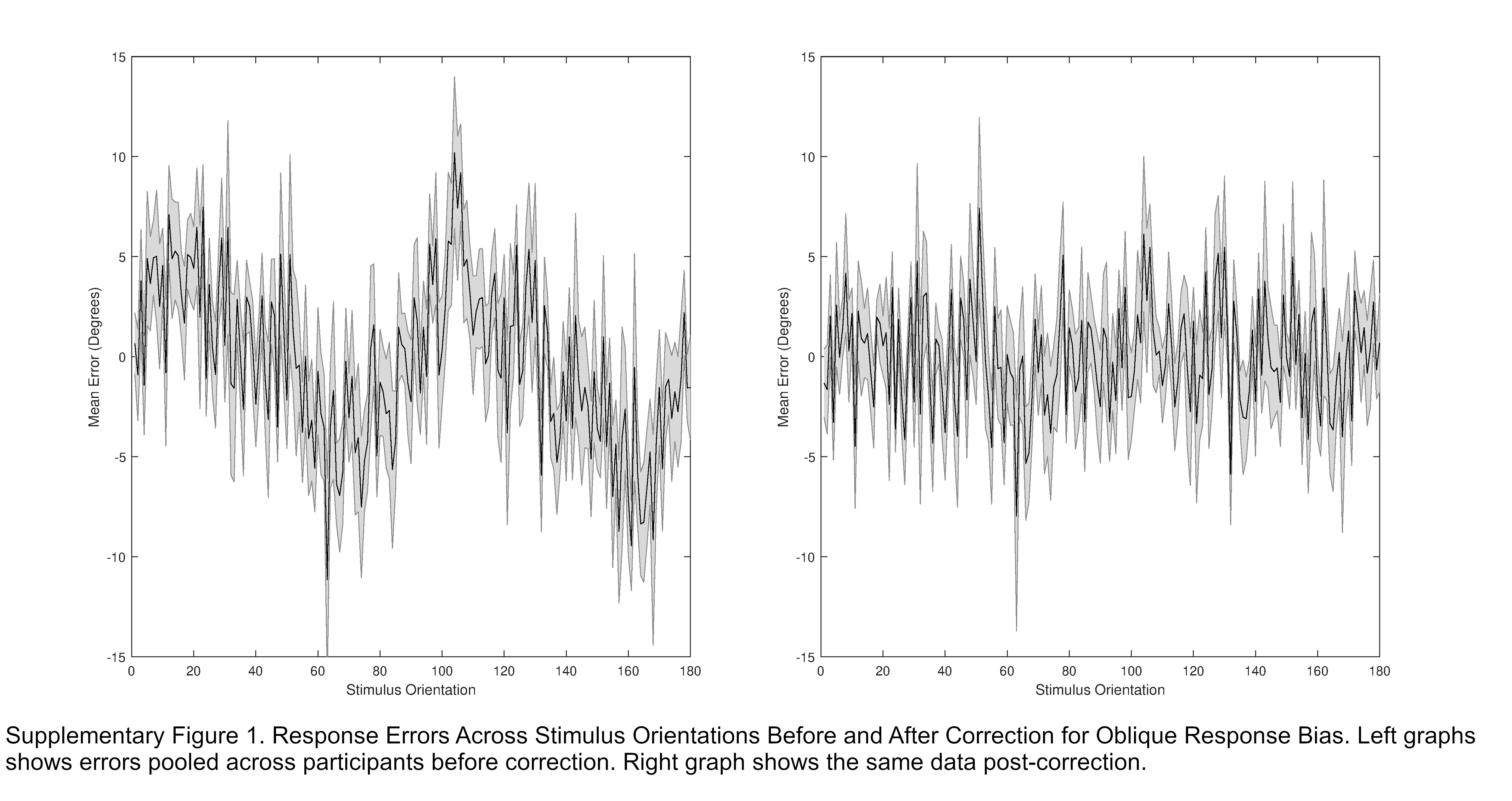

Supplement: Supplement 1 [file jovi-22-1-6_s001.jpg]
